# The potential of the solitary parasitoid *Microctonus brassicae* for the biological control of the adult cabbage stem flea beetle, *Psylliodes chrysocephala*

**DOI:** 10.1111/eea.12910

**Published:** 2020-05-15

**Authors:** Anna Jordan, Gavin R. Broad, Julia Stigenberg, Jessica Hughes, Jake Stone, Ian Bedford, Steven Penfield, Rachel Wells

**Affiliations:** ^1^ John Innes Centre Norwich Research Park Colney Lane Norwich Norfolk NR4 7UH UK; ^2^ Natural History Museum London UK; ^3^ Swedish Museum of Natural History Stockholm Sweden

**Keywords:** oilseed rape, *Brassica napus*, CSFB, parasitoid wasp, molecular barcode, *CO1*, Coleoptera, Chrysomelidae, Hymenoptera, Braconidae, biocontrol agent, Brassicaceae

## Abstract

The cabbage stem flea beetle (CSFB), *Psylliodes chrysocephala* L. (Coleoptera: Chrysomelidae), is a major pest of oilseed rape, *Brassica napus* L. (Brassicaceae), within the UK and continental Europe. Following the withdrawal of many broad‐spectrum pesticides, most importantly neonicotinoids, and with increased incidence of pyrethroid resistance, few chemical control options remain, resulting in the need for alternative pest management strategies. We identified the parasitoid wasp *Microctonus brassicae* (Haeselbarth) (Hymenoptera: Braconidae) within CSFB collected from three independent sites in Norfolk, UK. Parasitism of adult CSFB was confirmed, and wasp oviposition behaviour was described. Moreover, we show that within captive colonies parasitism rates are sufficient to generate significant biological control of CSFB populations. A sequence of the *M. brassicae* mitochondrial cytochrome oxidase 1 (*MT‐CO1*) gene was generated for rapid future identification. Moroccan specimens of *Microctonus aethiopoides* (Loan), possessing 90% sequence similarity, were the closest identified sequenced species. This study represents the first description published in English of this parasitoid of the adult cabbage stem flea beetle.

## Graphical abstract

The cabbage stem flea beetle (CSFB), *Psylliodes chrysocephala* (Coleoptera: Chrysomelidae), is a major pest of oilseed rape, *Brassica napus* (Brassicaceae), within Europe. Following the withdrawal of many broad‐spectrum pesticides, most importantly neonicotinoids, and with increased incidence of pyrethroid resistance, few chemical control options remain, resulting in the need for alternative approaches. Here we describe *Microctonus brassicae* (Hymenoptera: Braconidae), a parasitoid of adult CSFB, and show within captive colonies parasitism rates offer the potential to generate biological control of CSFB populations.

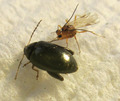

## Introduction

The cabbage stem flea beetle (CSFB), *Psylliodes chrysocephala* L. (Coleoptera: Chrysomelidae), is a major pest of oilseed rape (OSR), *Brassica napus* L. (Brassicaceae), particularly the winter crop within the UK and continental Europe (Alford & Gould, 1975; Graham & Alford, 1981; Bromand, 1990; Winfield, 1992; Alford et al., 2003; Williams, 2010). Measuring 3–5 mm in length, adult beetles are usually black with a metallic green/blue sheen, although there are brown variants (*Anglica*) (Hubble, 2010); they have pronounced hind femora enabling them to jump effectively, and they have rudimentary wings meaning they are also capable of flight (Hubble, 2010; Williams, 2010). Adult CSFB feed mainly on the leaves of Brassicaceae members, producing characteristic damage known as ‘shot‐holing’ (Ellis, 2015). This damage is most detrimental to seedlings, when the cotyledons, shoot apical meristem, stem, and first true leaves are attacked, often resulting in crop failure or poor crop establishment (Walters et al., 2001). The CSFB larvae feed within the stems and petioles causing more prolific damage, and if at a high enough density, this can result in plant death, early senescence, and significant reductions in yield (Lane & Cooper, 1989; Williams, 2004, 2010; Nicholls, 2016).

The CSFB has become a prominent pest in the UK, particularly in East Anglia and surrounding counties, following the 2013 EU moratorium on neonicotinoid seed treatment use in flowering crops (Wynn et al., 2014; Collins, 2017; Dewar, 2017; Kathage et al., 2017). Neonicotinoids are systemic pesticides which, when ingested, target the insect’s nervous system triggering intensified neuron stimulation and ultimately can lead to the death of the insect (Simon‐Delso et al., 2015; Bass & Field, 2018). The moratorium, based on a risk assessment of the European Food Safety Authority (EFSA, 2012), followed research linking the decline of beneficial insects, most importantly the honey bee, to the use of neonicotinoids in bee‐attractive crops, including maize, oilseed rape, and sunflower (van der Sluijs et al., 2013; Tsvetkov et al., 2017; Woodcock et al., 2017). In 2019, this moratorium was upgraded to a complete ban on the use of imidacloprid, clothianidin, and thiamethoxam on all crops from the end of 2018 (European Commision, 2013, 2018). With the subsequent removal of seed treatments for OSR, the numbers of CSFB and the damage they cause have increased (Collins, 2017). Within the UK in 2014, the Agriculture and Horticulture Development Board (AHDB) valued damage from CSFB at £23 million, with an approximate loss of 3.5% of the national crop area of winter OSR (Nicholls, 2016). Since the moratorium, chemical control for CSFB has been provided by foliar pyrethroid application but increasing incidences of resistance to this insecticide have been reported across Europe (Højland et al., 2016; Dewar, 2017). Such instances have highlighted the requirement for alternative pest control methodologies (Williams, 2010).

Within Europe, five species of parasitoid wasps (Hymenoptera) have been found to target CSFB: two ichneumonids, *Tersilochus microgaster* (Szépligeti) and *Aneuclis melanaria* (Holmgren), two braconids, *Diospilus morosus* (Reinhardt) and *Diospilus oleraceus* (Haliday), and one pteromalid, *Trichomalus lucidus* (Walker) (Ulber & Williams, 2003; Ulber et al., 2010). All species have been detected in the UK and Europe (de Jong et al., 2014). *Tersilochus microgaster* is well studied within the UK; however, the level of parasitism for this species in the field recorded in 2000 was estimated at around 10.8%, resulting in limited control efficacy (Ferguson et al., 2006; Williams, 2006). *Microctonus melanopus* (Ruthe) is the only species studied as a parasitoid of the adult beetle (Ulber et al., 2010). A solitary multivoltine endoparasitoid, it was reared from adults of the CSFB in the UK and also in France by Jourdheuil (1960) and later by Ulber & Williams (2003). Further description of the biology of this parasitoid is unavailable and the specimens cannot be traced. Four specimens of an additional species which emerged from adult CSFB collected by AW Fergerson of Rothamsted Research in Harpenden (Hertfordshire, UK) in 1996, were described by Haeselbarth in 2008 as a new species, *Perilitus brassicae* Haeselbarth. Haeselbarth (2008) regarded *Microctonus* as a synonym of *Perilitus*; however, Stigenberg et al. (2015) recommended that *Microctonus* should be recognised as a separate genus. *Perilitus brassicae* was transferred to *Microctonus* by Broad et al. (2016) and now named *Microctonus brassicae* (Haeselbarth). There have been no further specimens of this species reported until this study.

*Microctonus brassicae* is a univoltine imaginal endoparasitoid. Only solitary emergence from a host has been noted, although some species of *Microctonus* are known to be gregarious (summarised by Shaw & Huddleston, 1990). Once an egg has been laid, the beetle continues to feed as the larva develops within. When the parasitoid larva has passed through its final instar – thought to be the fifth, as with the majority of this family (Quicke, 2015) – the larva leaves the host’s body through the anus. It has not been determined whether the host dies before or after the larva emerges, but we anticipate it is the latter. The yellow hymenopteriform (maggot‐like) larva then seeks a protected site to pupate, spinning a silken pupal case around itself from which the adult wasp emerges at eclosion. Though feeding of the adult beetle is unlikely to be highly affected in the earlier stages of parasitism, the consumption of haemolymph will affect the behaviour of the beetle in the later stages, and hosts are probably quickly rendered sterile, as in other species of *Microctonus* (Loan & Holdaway, 1961; Drea, 1968).

*Microctonus* species of parasitoid wasps belong to the Braconidae, one of the largest families within Hymenoptera (Coşkun & Hikmet, 2011; Quicke, 2014). Some *Microctonus* species have already been introduced as classical biocontrol agents to control pest weevil species within the field (Aeschlimann, 1983). *Microctonus aethiopoides* Loan has been introduced into New Zealand as a classical biocontrol agent on farms growing large areas of pasture, dominated by *Medicago* sp. These introductions have varied in the ability to control the several pest weevil species present (Cullen & Hopkins, 1982); however, one target weevil species, *Sitona discoideus* (Gyllenhal), was found to have maintained a parasitism level of approximately 50% 9 years after introduction (Kean & Barlow, 2000). *Microctonus hyperodae* Loan was released in New Zealand for biological control of Argentine stem weevil, *Listronotus bonariensis* (Kuschel), a significant pest of graminaceous plants (Prestidge et al., 1991). After 12 months, parasitism levels ranged from 1.2% for Canterbury (South Island) to an average of 52% for three sites at Murapara (North Island) (Barlow et al., 1994). Differences in parasitism were attributed to climate, with greater parasitism levels occurring in a warmer environment (Barlow et al., 1994).

Here, we present *M. brassicae*, a parasitoid wasp of the adult CSFB, collected in 2017 within host CSFB from three independent sites in Norfolk, UK. Prior to this study *M. brassicae* was a largely unknown species, described in German and with limited anatomical illustrations available for identification. We provide detailed descriptions and illustrations to allow the identification of this parasitoid and separation from other *Microctonus* species. Here we also provide sequence data of the *M. brassicae* mitochondrial cytochrome oxidase 1 (*MT‐CO1*) gene which will facilitate rapid identification and aid future studies. Furthermore, we detail the wasp lifecycle, behaviour, oviposition, and parasitism of adult CSFB within captive colonies, providing the first full description of the interaction between *M. brassicae* and its host.

## Materials and methods

### Collection and culturing of field‐collected flea beetles

Approximately 3 000 adult CSFB were collected from three UK field sites within Norfolk, UK: Fincham (July 2017), Carleton Rode (July to September 2017), and Wymondham (September 2017). Following harvest, samples were collected from harvested material, leaf litter in gardens adjacent to harvested fields, and field margin weeds and leaf litter. To best allow natural behaviour, beetles were maintained on potted OSR (varieties Temple and Matador) or pak choi, *Brassica rapa* subsp. *chinensis* (L.) Hanelt (cv. China Choi), within micro‐perforated bags (38 × 90 cm; Focus Packaging, Wolverhampton, UK) in controlled environment rooms at L16(22 °C day):D8(20 °C) photo‐thermoperiod. During this time, parasitoids were discovered within captive colony bags, and initial oviposition behaviour within a colony was observed over a number of hours. Beetle colonies were checked daily for parasitoid emergence.

### Maintenance of emerged parasitoids

Upon emergence wasps were transferred to single sex colonies in 50‐ml Corning tubes. Several wasps were also maintained as individuals to observe unmated longevity. Wasps were provided with food (cotton wool soaked with a 1:1 mix of honey and water solution) and maintained within a controlled environment room at L14(22 °C day):D10(20 °C) photo‐thermoperiod. Wasp specimens were also collected and preserved in 100% ethanol for later identification and molecular analysis.

### Taxonomic identification of *Microctonus brassicae*

Identification was carried out at the Natural History Museum (London, UK; NHMUK) using a key by Haeselbarth (2008), comparing multiple specimens of each sex with the *Microctonus* species represented in the collections. Figures within this paper (except Figure 2) are all based on specimens within collections of the NHMUK. Scanning electron micrographs of uncoated specimens were taken using a JEOL IT500 SEM under variable pressure mode. Photographs were taken using a Canon SLR EOS 5DSR with 65‐mm macro lens mounted on a copy stand with an automated Z‐stepper; images were aligned using Helicon Focus software v.6.6.1 (Helicon Soft, Kharkiv, Ukraine). Specimens were directly compared to *Microctonus debilis* (Waterston) and *Microctonus sommerae* (Haeselbarth) as species most likely to be confused with *M. brassicae*. Specimens were deposited in NHMUK, Royal Museum of Scotland (Edinburgh), Swedish Museum of Natural History (Stockholm), and Naturalis Biodiversity Center (Leiden).

### Molecular characterisation of *Microctonus brassicae* via *MT‐CO1*

Parasitoid DNA was extracted from single ethanol‐preserved specimens following the protocol from the DNeasy Tissue Kit (Qiagen, Valencia, CA, USA). A 658‐bp fragment from the 5′ region of *MT‐CO1* was amplified using the LCO (light DNA strand CO1) and HCO (heavy DNA strand CO1) primers (Folmer et al., 1994) using Ready‐To‐Go PCR beads (Amersham Pharmacia Biotech, Amersham, UK) on the following program: 5 min 94 °C hotstart; 40 cycles: denature 94 °C for 15 s, anneal 46 °C for 15 s, extend 72 °C for 15 s; final extension 72 °C for 10 min. This gene has been used in previous studies of braconid phylogenetics (Belshaw et al., 2000; Belshaw & Quicke, 2002; Dowton et al., 2002; Zaldivar‐Riverón et al., 2006; Sharanowski et al., 2011; Stigenberg & Ronquist, 2011). Product yield and specificity were observed using agarose gel electrophoresis. PCR products were purified with EXO1 and FastAP. The product was sequenced using both the forward and reverse primers and sequencing reactions were purified with the DyeEx 96 kit (Qiagen). Sequences were assembled and edited using Geneious Pro v.9.1.8. The Voseq v.1.7.3 (Peña & Malm, 2012) database was used for storing voucher and DNA sequence data. BLAST analysis was performed to identify similar sequences within the NCBI database.

### Lifecycle, behaviour, and parasitism of adult CSFB by *Microctonus brassicae*

Unrelated male and female wasps derived from beetle colonies from the various collection sites were moved into 50‐ml Corning tubes for 24 h to allow successful mating before they were transferred into ventilated Perspex experiment boxes (60 × 115 × 175 mm), lined with damp tissue paper and containing young adult CSFB and two Chinese cabbage leaves, *Brassica rapa* subsp. *pekinensis* (L.) Hanelt var. Hilton, as a beetle food source. Chinese cabbage leaves were changed once per week. Wasps were provided with food solution via 1:1 honey–water soaked blue roll within a 2‐cm‐diameter Petri dish lid. The boxes were kept condensation free by the removal of moisture with paper towels. Experiments were conducted through time with variable wasp and beetle numbers in each experiment depending on insect availability. One to three female wasps and 10‐45 CSFB were used per experiment. Wasps were observed at room temperature for behavioural tendencies including host selection and oviposition behaviour for a minimum of 20 min for each of the experimental runs on transfer to Corning tubes and directly after setting up the experiment boxes. Following host death, CSFB cadavers were kept on damp blue roll to avoid larval desiccation and promote the production of viable pupae. Pupae and larvae are delicate therefore CSFB cadavers were left in situ, allowing the larvae to evacuate and pupate within the box with minimal disturbance. The time to complete lifecycle stages and the numbers of larvae, pupae, and adult wasps were recorded. Parasitism rate and wasp sex ratio were calculated. Longevity of unmated males and females were recorded and statistical differences between sexes were analysed with t‐tests.

## Results

### Description and identification of *Microctonus brassicae*

Large numbers of adult wasps were first detected within colonies of beetles maintained under laboratory conditions approximately 2 months after collection. Over 60 wasps were used for experimentation and identification but many more were obtained over this period. Wasp presence resulted in beetle colony collapse if parasitoids were not removed (approximately 100 beetles per colony). Parasitoids had not been observed within captive colonies prior to this; however, previous generations of CSFB had been reared from plants containing larvae and not adult beetles. Adult beetles have previously been collected for chemical resistance testing but are not maintained for long periods or used for rearing colonies, thus explaining a lack of previous observations.

**Figure 1:**
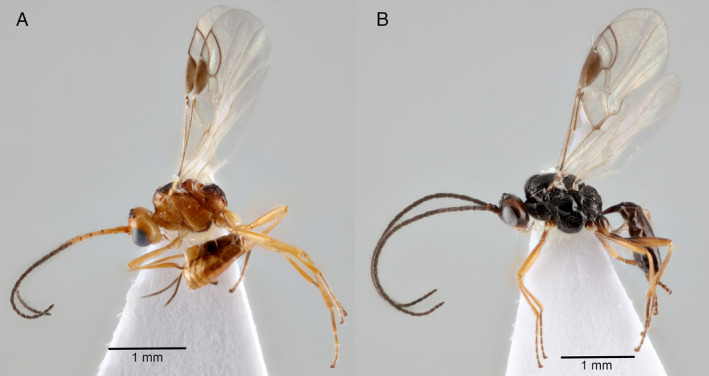
(A) Female and (B) male *Microctonus brassicae* reared from *Psylliodes chrysocephala* collected in Norfolk, UK. [Colour figure can be viewed at wileyonlinelibrary.com]

**Figure 2:**
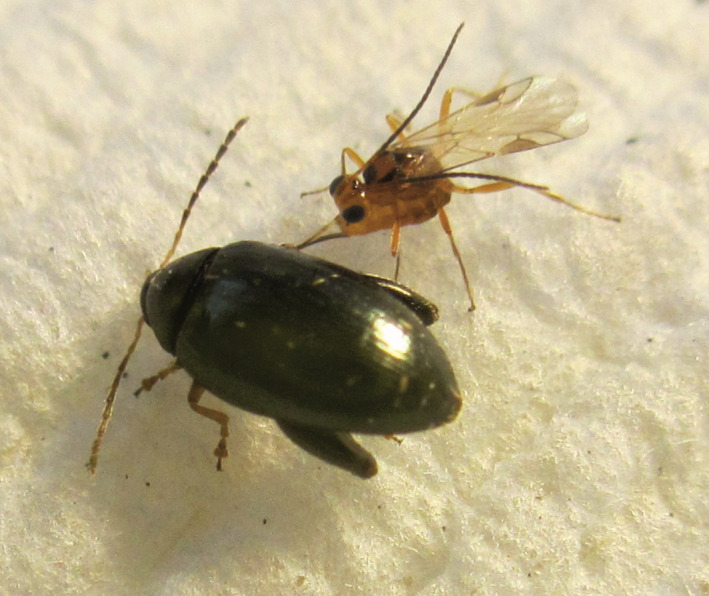
Female *Microtonus brassicae* alongside an adult *Psylliodes chrysocephala*, ovipositing between the beetle’s pronotum and elytrum. [Colour figure can be viewed at wileyonlinelibrary.com]

The wasps were identified to be sexually dimorphic, with the females being orange/brown (Figure 1A) and the males mainly black with yellow/brown legs (Figure 1B). Females could be identified from oviposition behaviour (Figure 2). Female body length ranged from 2–2.5 mm from head to tip of metasoma (excluding antennae and ovipositor) with an mean body length of 2.3 mm (n = 10). Male body length ranged from 2–3 mm from head to tip of metasoma (excluding antennae) with an mean body length of 2.6 mm (n = 10). Estimation of body length for 361 species of parasitoid wasps from 21 families by Hurlbutt (1987) showed that in most species, females are generally larger than males. As in our study, however, species in the family Ichneumonidae (subfamily Ichneumoninae) were different, with males generally significantly longer than females. However, as pointed out by Gauld & Fitton (1987), body length is a poor measure of overall size and mass in parasitoid wasps, with the few available studies on Ichneumonidae demonstrating that females have a greater overall mass, and usually a longer wing length.

**Figure 3:**
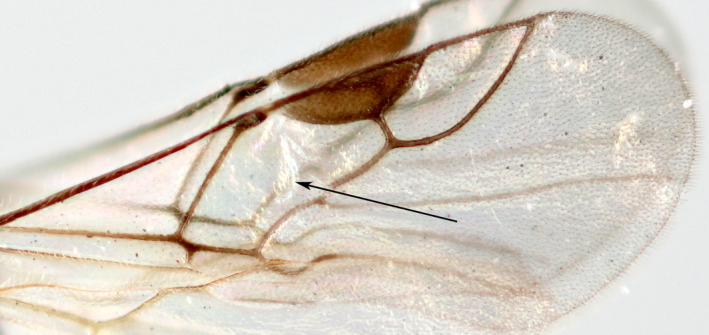
*Microtonus brassicae* forewing. The arrow points to discosubmarginal cell (combined first discal and first submarginal cells). [Colour figure can be viewed at wileyonlinelibrary.com]

Species lacking forewing vein 1‐SR+M, and consequently possessing a large discosubmarginal cell (Figure 3), were listed as species of *Microctonus* by Broad et al. (2016). This is a large genus with at least 27 known British and Irish species. The parasitoid wasp was identified from multiple specimens as *M. brassicae*. No other CSFB parasitoids were observed to emerge from the adults.

**Figure 4:**
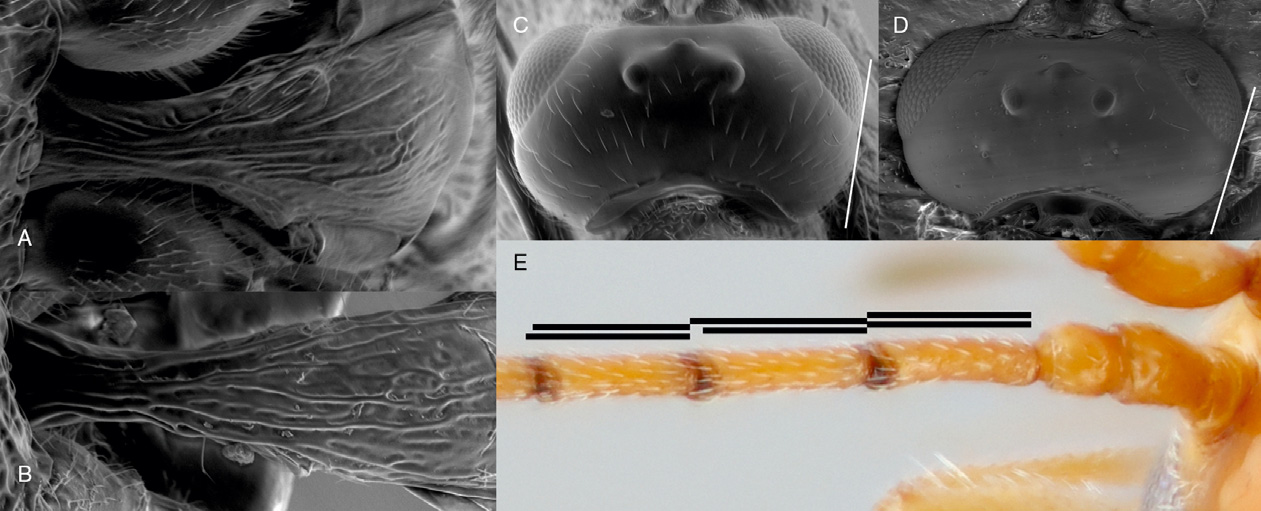
First metasomal tergite, dorsal: (A) *Microtonus brassicae* female; (B) *M. sommerae* female. Head, dorsal: (C) *M. brassicae* female; (D) *M. debilis* female. The white line demonstrates the degree to which the temples are narrowed behind the eyes (line passing the outermost parts of the eye and temple). (E) Proximal antenna segments of female *M. brassicae*, showing relative lengths of first, second, and third flagellar segments. Uppermost lines represent the actual lengths of the segments, lower lines represent the length of the first flagellar segment. [Colour figure can be viewed at wileyonlinelibrary.com]

**Figure 5:**
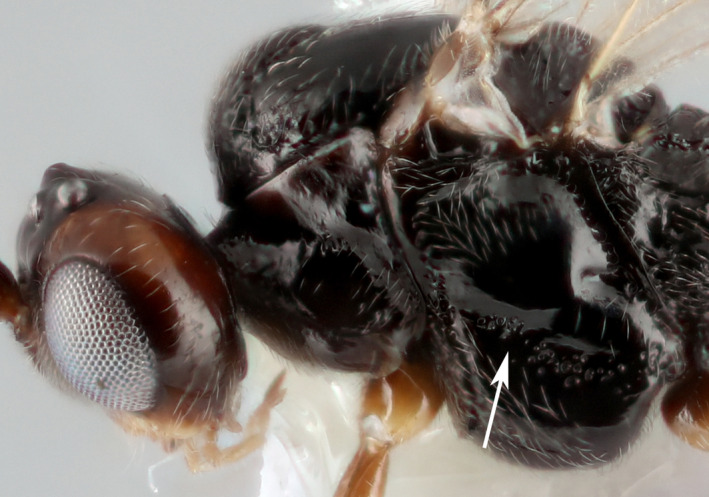
Mesopleuron of male *Microtonus brassicae*. The arrow points to the narrow precoxal sulcus. [Colour figure can be viewed at wileyonlinelibrary.com]

The female ovipositor sheath is narrow and straight and the first metasomal tergite and sternite are separated. The colour pattern of females is distinctive (Figure 1A): the body is basically pale red with three large black marks on the mesoscutum, the propodeum is mostly black, and the antenna is abruptly darker from the fifth flagellar segment distally. Males are very different, mostly black but with a large area of dull red on the head (malar space, vertex, and inner orbit; Figure 1B) and a pale clypeus. *Microctonus brassicae* is also characterised by its elongate first metasomal tergite, lacking dorsopes (Figure 4A,B); rounded temples (Figure 4C,D); the first flagellar segment of the antenna is slightly shorter than the second, the third is much shorter (Figure 4E); occipital carina complete but weak medially (Figure 4C,D); hind coxa unsculptured, and precoxal sulcus of the mesopleuron narrow (Figure 5).

**Figure 6:**
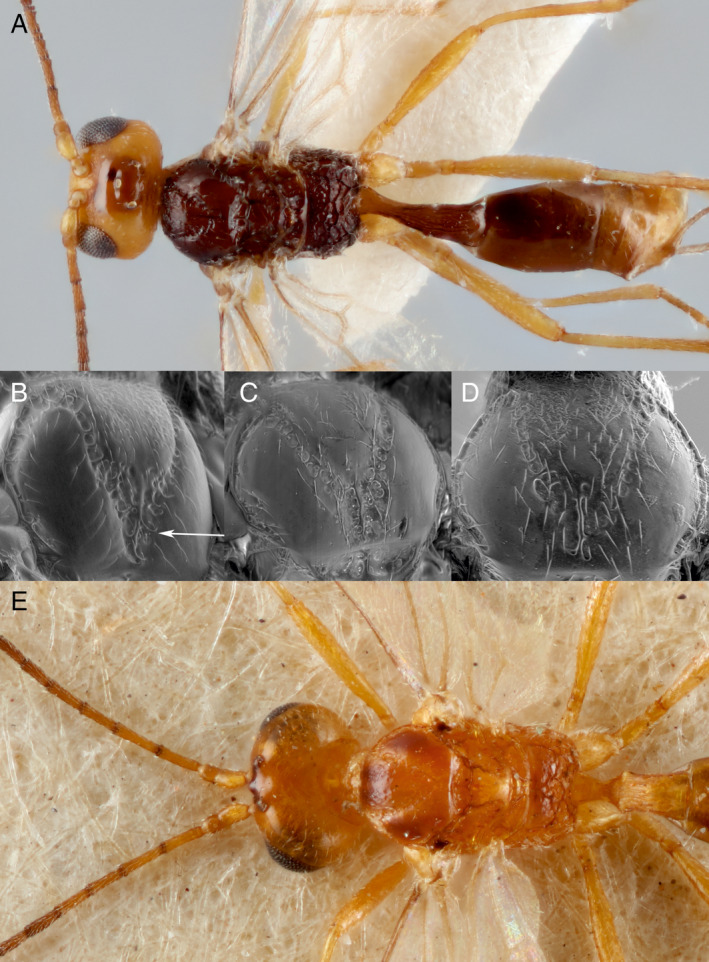
(A) *Microctonus sommerae* paratype female, dorsal. Mesoscutum: (B) *M. brassicae* (arrow points to elongate area of rugosity); (C) *M. debilis*; (D) *M. sommerae*. (E) *Microctonus debilis* paratype female, dorsal. [Colour figure can be viewed at wileyonlinelibrary.com]

Identification of *M. brassicae* using Haeselbarth’s (2008) key to European species can be complicated by the strong sexual dimorphism and the fact that females do not run satisfactorily from Haeselbarth’s couplet 43, because of the unclear distinction between the length of the second and third flagellar segments. *Microctonus sommerae* and *M. debilis* are the species most likely to be confused with *M. brassicae* when running specimens through Haeselbarth’s key, therefore we provide direct comparison with these species to aid identification. *Microctonus sommerae* is structurally similar but differs in colour pattern (Figure 6A) and in the sculpture of the first metasomal tergite, which has some transverse rugae mixed with the longitudinal striae, whereas *M. brassicae* has a simpler sculpture of sparse longitudinal striae (Figure 4A,B). The shape of the posterior rugose area on the mesoscutum also differs between species (Figure 6B–D), this area being particularly long and triangular in *M. brassicae*. *Microctonus sommerae* has not yet been found in Britain but has been reared from *Phyllotreta* species (Coleoptera: Chrysomelidae) in several other European countries (Haeselbarth, 2008).

As noted by Haeselbarth (2008), *M. brassicae* is rather similar to *M. debilis* (Figure 6E). The colour pattern of *M. debilis* is different, with the black markings paler and ill‐defined and the antenna fading from yellow to black, rather than the colour abruptly changing. Females of *M. brassicae* seen so far have 21 flagellar segments, *M. debilis* usually has 18 or 19 (ranging from 16 to 21 according to Haeselbarth), and the temples are more abruptly narrowed behind the eyes in *M. debilis* than in *M. brassicae* (Figure 4C,D).

Males of *M. brassicae* have longer antennae than females, with 25 or 26 flagellar segments. Both sexes have a long, slender first tergite, compared to most other *Microctonus*, and the precoxal sulcus is narrow, rather than a wide area of rugosity (Figure 6B–D).

### Identification of wasp and molecular characterisation

Sequencing of the 658 bp 5′ end of *MT‐CO1* (GenBank: MH790287) and subsequent BLAST analysis (Table S1) identified 93% sequence similarity to two unclassified braconid Euphorinae identified in a Canadian study (Hebert et al., 2016). A 90% sequence similarity was observed to Moroccan specimens of *M. aethiopoides* and 89% to *M. hyperodae* from New Zealand (Vink et al., 2012). No records were present for *M. brassicae*, identifying this as the first molecular characterisation of this species and the first full description of a parasitoid of the adult CSFB.

### Lifecycle and behaviour of *Microctonus brassicae* within captive colonies of CSFB

Male unmated wasps, when fed on a 1:1 mix of honey and water solution, lived on average 14.1 days (range: 5–22; n = 8), females 8.4 days (range: 4–11; n = 5) (Table S2). However, this difference in longevity was not statistically significant (t‐test: P = 0.08).

Female *M. brassicae* mated once per individual male. If housed with multiple males, each mated in turn with the female. Females oviposited whether mated or not. As with other Hymenoptera, *M. brassicae* are haplodiploid, unfertilised eggs producing only male offspring (Quicke, 2015). Females stalked potential hosts from behind before attempting to oviposit. Active beetles were preferred as hosts. If a beetle being stalked became stationary, the female switched to a new, more mobile host. Wasps attempted to oviposit in several areas of the host’s body, including the mouthparts and the tip of the abdomen. The preferred site of oviposition was between the pronotum and elytra (Figure 2). Wasps made multiple attempts to oviposit, but only a single successful oviposition event was observed per host. Following successful oviposition, wasps no longer interacted with hosts.

*Microctonus brassicae* is a solitary koinobiont endoparasitoid. An egg is laid within the host and the larva develops within the host’s body. Once parasitized, the host beetle continued to feed and exhibit normal behaviour up until the parasitoid was almost ready to leave the host. Examination of the beetle cadavers showed no exit holes. The larvae are therefore assumed to exit through the anus of the beetles. Larvae varied in size with a mean body length of 2.5 mm (n = 10). Once larvae exited the host, they spun silken cocoons. These pupae varied in size, with the smallest pupa collected measuring 2 mm in length. This failed to develop, however, presumably due to its small size. Most pupae collected ranged from 2.5–3 mm (n = 10), with a mean length of 3 mm. Adult wasps eclosed after an average of 11.9 days. The time taken to complete the whole lifecycle, from oviposition in the CSFB to eclosion of the adult, took on average 43.5 days (Table 1).

**Table 1:** Fertility and parasitism rate of the parasitoid wasp *Microctonus brassicae* across 10 controlled mating and parasitism trials. Unrelated male and female wasps derived from *Psylliodes chrysocephala* beetle colonies from three collection sites were contained together for 24 h to promote mating before being added to experimental boxes containing variable numbers of beetles

Experimental set up					Experimental results								
Experimental run		No. CSFB	No. wasps added		Emerged larvae	Emerged pupae	Emerged adults	Parasitism rate (%)	No. wasps eclosed		Sex ratio (% ♀)	Development time (days)	
No.	Date		♀	♂					♀	♂		Egg to pupae	Pupae to adult
1	22/12/2017	25	1	4	12	11	6	48	6	0	100	27	10
2	10/01/2018	32	3	5	15	11	1	46.88	0	1	0	52	14
3	17/01/2018	10	1	1	5	3	2	50	1	1	50	34	14
4	29/01/2018	27	1	0	12	11	11	44.44	2	9	18.18	29	13
5	29/01/2018	15	1	1	8	8	6	53.33	0	6	0	30	17
6	27/02/2018	34	1	1	15	14	13	44.12	9	4	69.23	28	13
7	12/03/2018	30	1	2	16	14	11	53.33	5	6	45.45	26	11
8	14/03/2018	20	1	1	8	8	8	40	0	8	0	27	9
9	09/04/2018	23	2	1	6	6	2	26.09	0	2	0	38	7
10	09/04/2018	45	1	1	15	7	7	33.33	3	4	42.86	25	11
Mean ± SD								43.95 ± 8.28			32.75 ± 33.04	31.6 ± 7.76	11.9 ± 2.74

### Wasp fertility and parasitism rate

Overall, average wasp parasitism rate for CSFB infestation within captivity was 44% (Table 1). To determine whether the variable numbers of female parasitoids and hosts influenced parasitism rate across experiments, we have compared experimental runs. In instances where a single female parasitoid was used with varying numbers of host beetles, parasitism level is on average 46% across experiments. The first experiment with multiple females (run 2) has a similar parasitism rate of 47%. Run 9, containing multiple females, and run 10, containing a single female, both performed poorly compared to other runs, with parasitism rates of 26 and 33%, respectively. The parasitoids used within experiments would have been freshly emerged; however, beetle age will have increased across the duration of the experiment. This may indicate there is an association between beetle age and suitability as a host as selected by the wasp.

The percentage of larvae that successfully developed into pupae was high, on average 84%, indicating low mortality during this transition. The percentage of pupae that successfully eclosed into adults was slightly reduced (71%), indicating that even if larvae pupate successfully, the pupae may not go on to produce viable adults. Whether this is a result of natural mortality or the captive rearing environment is unknown. On average, 33% of the adults to eclose were female, with some experiments producing 100% male offspring, presumably because these females had not successfully mated.

## Discussion

With the withdrawal of the neonicotinoid class of insecticides the CSFB has become a major pest of winter OSR, with increases in pest incidence reported across the UK and continental Europe (Kathage et al., 2017; Scott & Bilsborrow, 2019). Changes in weather patterns brought about from climate change may result in a more unpredictable UK climate and milder than average temperatures throughout the winter (Lowe et al., 2019). These conditions potentially allow adult beetles to survive for longer, laying more eggs, resulting in higher larval load in the crops (Collins, 2017). The parasitoid *M. brassicae* could be highly beneficial in the control of CSFB, especially with resistance to the remaining pyrethroid pesticides now detected in the beetles across Europe (Højland et al., 2015; Robert et al., 2017; Stará & Kocourek, 2019).

Only two UK populations of *M. brassicae* have been found so far, in Harpenden (the type series of the species) and in Norfolk, as presented here. That these populations are so clearly geographically separated may indicate that the wasps are more widespread than we are currently aware of. Determining the geographical distribution of the wasp, field parasitism rates, and environmental factors affecting parasitism will be of considerable interest.

Studies on the biological control of *S. discoideus* by *M. aethiopoides* showed 54% parasitism in controlled laboratory experiments (Barratt & Johnstone, 2001). A field parasitism level of approximately 50% was maintained 9 years after introduction as a control agent (Kean & Barlow, 2000). As a result, insecticidal control was usually found to be economically unnecessary (Barlow & Goldson, 1993). The ca. 44% parasitism rate of *M. brassicae* in captivity suggests that the parasitoid may have the potential to deliver positive effects under field conditions. Our research has shown that it is possible to rear captive *M. brassicae* and has detailed the lifecycle of *M. brassicae* in laboratory conditions. The generation time of 43.5 days (from oviposition to adult emergence) within controlled conditions means it would be possible to rapidly rear multiple generations, given the collection/maintenance of successful captive host colonies. It is unclear whether this could be successfully undertaken on a larger scale for the purposes of inundative (augmentative) biological control. For *M. aethiopoides*, availability of the host *S. discoideus* was dependent on field‐collected material as it was impossible to rear sufficient weevils in captivity. Imported weevils were used as hosts when no native weevils were present (Cullen & Hopkins, 1982).

Though we have successfully established methodologies for the successful rearing of captive CSFB this is currently slow and costly. Developing systems to supress aestivation, for example by photoperiod or temperature manipulation, would be necessary to produce rapid cycling colonies. Cabbage stem flea beetle can be collected in large numbers at harvest within the combine hopper. Depending on UK distribution, adults from areas shown to have positive infestation could be released in other areas.

*Microctonus brassicae* is arrhenotokous, with unfertilised eggs developing into males. Sterile‐diploid males may also be produced from loss of complimentary sex determination alleles (CSD) during inbreeding. Interestingly, *M. aethiopoides* from Ireland, as opposed to other areas of Europe, is thelytokous, therefore fertilisation is not required for the production of female wasps (McNeill et al., 2006). Whether a similar phenomenon exists in *M. brassicae* is unknown. Study of independent populations will be required.

Concentrating efforts on conservation biocontrol, i.e., protecting and boosting the wild populations of *M. brassicae*, would support integrated pest management approaches. Further work would be needed to study the lifecycle of the parasitoid wasp within its natural environment. With an increased knowledge of the wild populations, agricultural management strategies could be adjusted to aid parasitoid survival and promote an increase in the native population. Mechanical cultivation and chemical application, which may disturb and damage the various life stages, could be timed or carried out in a way that is least likely to affect the wasps (Gillespie et al., 2016; Skellern & Cook, 2018). The promotion of field margins, beetle banks, and conservation headland management may provide habitat to support and protect parasitoids (Holland et al., 2016). Other hosts of *M. brassicae* are unknown, therefore wasp numbers present within the field may be directly dependant on CSFB numbers. As in the Lotka‐Volterra model, the wasps’ abundance at different time points will relate to how abundant the host is over that period (Hassell, 2000). Successful control of CSFB may result in a reduction in wasp numbers due to a decline in host availability. Supporting a balance of parasitoid to host will be key in the exploitation of natural controls.

Given the challenges being encountered with the declining numbers of pesticides now available, supporting natural biocontrol methods is likely to play an ever more vital role in controlling major crop pests. Identifying novel species and developing management strategies to exploit beneficial insects will be of increasing importance for future crop protection.

## Supporting information


**Table S1.** Top 10 BLAST hits of *Microctonus brassicae* mitochondrial cytochrome oxidase 1 (*MT‐CO1*) gene sequence to the NCBI nucleotide database.
**Table S2.** Longevity of unmated male and female *Microctonus brassicae* wasps.
