# Supplementary material for: The potential of the solitary parasitoid Microctonus brassicae for the biological control of the adult cabbage stem flea beetle, Psylliodes chrysocephala
Source: Entomol Exp Appl. 2020 May 15;168(5):360–70. doi: 10.1111/eea.12910 (PMC7386932; doi:10.1111/eea.12910)
Supplement: Supplementary file 1 — Table S1. Top 10 BLAST hits of Microctonus brassicae mitochondrial cytochrome oxidase 1 (MT‐CO1) gene sequence to the NCBI nucleotide database. Table S2. Longevity of unmated male and female Microctonus brassicae wasps. [file EEA-168-360-s001.docx]

**The potential of the solitary parasitoid *Microctonus brassicae* for the biological control of the adult cabbage stem flea beetle,** ***Psylliodes chrysocephala***

**Anna Jordan^1^, Gavin R. Broad^2^, Julia Stigenberg^3^, Jessica Hughes^1^, Jake Stone^1^, Ian Bedford^1^, Steven Penfield^1^ & Rachel Wells^1^***

^1^John Innes Centre, Norwich Research Park, Colney Lane, Norwich, Norfolk, UK NR4 7UH, ^2^Natural History Museum, London, UK, and ^3^Swedish Museum of Natural History, Stockholm, Sweden

**Supplementary Tables**

| Hit | Description | [Score](https://blast.ncbi.nlm.nih.gov/Blast.cgi?CMD=Get&ALIGNMENTS=100&ALIGNMENT_VIEW=Pairwise&DATABASE_SORT=0&DESCRIPTIONS=100&DYNAMIC_FORMAT=on&FIRST_QUERY_NUM=0&FORMAT_OBJECT=Alignment&FORMAT_PAGE_TARGET=&FORMAT_TYPE=HTML&GET_SEQUENCE=yes&I_THRESH=&LINE_LENGTH=60&MASK_CHAR=2&MASK_COLOR=1&NUM_OVERVIEW=100&PAGE=MegaBlast&QUERY_INDEX=0&QUERY_NUMBER=0&RESULTS_PAGE_TARGET=&RID=6GP0D0TW014&SHOW_LINKOUT=yes&SHOW_OVERVIEW=yes&STEP_NUMBER=&OLD_VIEW=false&DISPLAY_SORT=1&HSP_SORT=1) | | [Query](https://blast.ncbi.nlm.nih.gov/Blast.cgi?CMD=Get&ALIGNMENTS=100&ALIGNMENT_VIEW=Pairwise&DATABASE_SORT=0&DESCRIPTIONS=100&DYNAMIC_FORMAT=on&FIRST_QUERY_NUM=0&FORMAT_OBJECT=Alignment&FORMAT_PAGE_TARGET=&FORMAT_TYPE=HTML&GET_SEQUENCE=yes&I_THRESH=&LINE_LENGTH=60&MASK_CHAR=2&MASK_COLOR=1&NUM_OVERVIEW=100&PAGE=MegaBlast&QUERY_INDEX=0&QUERY_NUMBER=0&RESULTS_PAGE_TARGET=&RID=6GP0D0TW014&SHOW_LINKOUT=yes&SHOW_OVERVIEW=yes&STEP_NUMBER=&OLD_VIEW=false&DISPLAY_SORT=4&HSP_SORT=0) | [Identity](https://blast.ncbi.nlm.nih.gov/Blast.cgi?CMD=Get&ALIGNMENTS=100&ALIGNMENT_VIEW=Pairwise&DATABASE_SORT=0&DESCRIPTIONS=100&DYNAMIC_FORMAT=on&FIRST_QUERY_NUM=0&FORMAT_OBJECT=Alignment&FORMAT_PAGE_TARGET=&FORMAT_TYPE=HTML&GET_SEQUENCE=yes&I_THRESH=&LINE_LENGTH=60&MASK_CHAR=2&MASK_COLOR=1&NUM_OVERVIEW=100&PAGE=MegaBlast&QUERY_INDEX=0&QUERY_NUMBER=0&RESULTS_PAGE_TARGET=&RID=6GP0D0TW014&SHOW_LINKOUT=yes&SHOW_OVERVIEW=yes&STEP_NUMBER=&DISPLAY_SORT=3&HSP_SORT=3) (%) | NCBI Accession |
| --- | --- | --- | --- | --- | --- | --- |
|  |  | Max | Total | coverage (%) |  | No. |
| 1 | [Euphorinae sp. BOLD-2016 voucher BIOUG16070-A01 cytochrome oxidase subunit 1 (COI) gene, partial cds; mitochondrial](https://blast.ncbi.nlm.nih.gov/Blast.cgi#alnHdr_1063179658) | 859 | 859 | 88 | 93 | [KR878896.1](https://www.ncbi.nlm.nih.gov/nucleotide/KR878896.1?report=genbank&log$=nucltop&blast_rank=1&RID=6GP0D0TW014) |
| 2 | [Euphorinae sp. BOLD-2016 voucher BIOUG16070-F04 cytochrome oxidase subunit 1 (COI) gene, partial cds; mitochondrial](https://blast.ncbi.nlm.nih.gov/Blast.cgi#alnHdr_1052465949) | 852 | 852 | 87 | 93 | [KR901665.1](https://www.ncbi.nlm.nih.gov/nucleotide/KR901665.1?report=genbank&log$=nucltop&blast_rank=2&RID=6GP0D0TW014) |
| 3 | [*Microctonus* sp. CJV-2012 isolate 10079 cytochrome c oxidase subunit 1 (COI) gene, partial cds; mitochondrial](https://blast.ncbi.nlm.nih.gov/Blast.cgi#alnHdr_384489570) | 845 | 845 | 100 | 90 | [JN980125.1](https://www.ncbi.nlm.nih.gov/nucleotide/JN980125.1?report=genbank&log$=nucltop&blast_rank=3&RID=6GP0D0TW014) |
| 4 | [*Microctonus aethiopoides* voucher BIOUG02434-C09 cytochrome oxidase subunit 1 (COI) gene, partial cds; mitochondrial](https://blast.ncbi.nlm.nih.gov/Blast.cgi#alnHdr_1214168351) | 832 | 832 | 99 | 89 | [KY845728.1](https://www.ncbi.nlm.nih.gov/nucleotide/KY845728.1?report=genbank&log$=nucltop&blast_rank=4&RID=6GP0D0TW014) |
| 5 | [*Microctonus aethiopoides* voucher BIOUG02399-F11 cytochrome oxidase subunit 1 (COI) gene, partial cds; mitochondrial](https://blast.ncbi.nlm.nih.gov/Blast.cgi#alnHdr_1214168116) | 832 | 832 | 99 | 89 | [KY845609.1](https://www.ncbi.nlm.nih.gov/nucleotide/KY845609.1?report=genbank&log$=nucltop&blast_rank=5&RID=6GP0D0TW014) |
| 6 | [*Microctonus aethiopoides* isolate 10069 cytochrome c oxidase subunit 1 (COI) gene, partial cds; mitochondrial](https://blast.ncbi.nlm.nih.gov/Blast.cgi#alnHdr_384489554) | 822 | 822 | 100 | 89 | [JN980117.1](https://www.ncbi.nlm.nih.gov/nucleotide/JN980117.1?report=genbank&log$=nucltop&blast_rank=6&RID=6GP0D0TW014) |
| 7 | [*Microctonus aethiopoides* isolate 10068 cytochrome c oxidase subunit 1 (COI) gene, partial cds; mitochondrial](https://blast.ncbi.nlm.nih.gov/Blast.cgi#alnHdr_384489552) | 821 | 821 | 98 | 90 | [JN980116.1](https://www.ncbi.nlm.nih.gov/nucleotide/JN980116.1?report=genbank&log$=nucltop&blast_rank=7&RID=6GP0D0TW014) |
| 8 | [*Microctonus aethiopoides* isolate HY9c cytochrome oxidase subunit 1 (COI) gene, partial cds; mitochondrial](https://blast.ncbi.nlm.nih.gov/Blast.cgi#alnHdr_159024218) | 821 | 821 | 99 | 89 | [EU078335.1](https://www.ncbi.nlm.nih.gov/nucleotide/EU078335.1?report=genbank&log$=nucltop&blast_rank=8&RID=6GP0D0TW014) |
| 9 | [*Microctonus aethiopoides* isolate HY6a cytochrome oxidase subunit 1 (COI) gene, partial cds; mitochondrial](https://blast.ncbi.nlm.nih.gov/Blast.cgi#alnHdr_159024220) | 815 | 815 | 99 | 89 | [EU078336.1](https://www.ncbi.nlm.nih.gov/nucleotide/EU078336.1?report=genbank&log$=nucltop&blast_rank=9&RID=6GP0D0TW014) |
| 10 | [*Microctonus aethiopoides* isolate 10130 cytochrome c oxidase subunit 1 (COI) gene, partial cds; mitochondrial](https://blast.ncbi.nlm.nih.gov/Blast.cgi#alnHdr_384489590) | 809 | 809 | 99 | 89 | [JN980135.1](https://www.ncbi.nlm.nih.gov/nucleotide/JN980135.1?report=genbank&log$=nucltop&blast_rank=10&RID=6GP0D0TW014) |

**Table S1** Top 10 BLAST hits of *Microctonus brassicae* mitochondrial cytochrome oxidase 1 (*MT-CO1*) gene sequence to the NCBI nucleotide database

| Sex | Wasp ID | Longevity (days) |
| --- | --- | --- |
| M | 1 | 13 |
|  | 2 | 22 |
|  | 3 | 16 |
|  | 4 | 7 |
|  | 5 | 11 |
|  | 6 | 17 |
|  | 7 | 5 |
|  | 8 | 22 |
| Mean ± SD |  | 14.12 ± 6.33 |
| Range |  | 5-22 |
| F | 1 | 7 |
|  | 2 | 4 |
|  | 3 | 10 |
|  | 4 | 10 |
|  | 5 | 11 |
| Mean ± SD |  | 8.4 ± 2.88 |
| Range |  | 4-11 |

**Table S2** Longevity of unmated male and female *Microctonus brassicae* wasps
